# Induction of the Endoplasmic-Reticulum-Stress Response: MicroRNA-34a Targeting of the IRE1α-Branch

**DOI:** 10.3390/cells9061442

**Published:** 2020-06-10

**Authors:** Lena Krammes, Martin Hart, Stefanie Rheinheimer, Caroline Diener, Jennifer Menegatti, Friedrich Grässer, Andreas Keller, Eckart Meese

**Affiliations:** 1 Institute of Human Genetics, Saarland University, 66421 Homburg, Germany; martin.hart@uks.eu (M.H.); stefanie.rheinheimer@uks.eu (S.R.); Caroline.Diener@uni-saarland.de (C.D.); eckart.meese@uks.eu (E.M.); 2 Institute of Virology, Saarland University, 66421 Homburg, Germany; jenny_m@web.de (J.M.); Friedrich.Graesser@uks.eu (F.G.); 3 Chair for Clinical Bioinformatics, Saarland University, 66123 Saarbrücken, Germany; andreas.keller@ccb.uni-saarland.de; 4 Department of Neurology and Neurological Sciences, Stanford University School of Medicine, Stanford, CA 94305, USA

**Keywords:** neurodegeneration, unfolded protein response, *BIP*, *IRE1α*, *XBP1*, miR-34a-5p, endoplasmic reticulum

## Abstract

Neurodegenerative disorders such as Alzheimer’s disease (AD) and Parkinson’s disease (PD) are characterized by the accumulation of misfolded proteins in the endoplasmic reticulum (ER) and the unfolded protein response (UPR). Modulating the UPR is one of the major challenges to counteract the development of neurodegenerative disorders and other diseases with affected UPR. Here, we show that miR-34a-5p directly targets the IRE1α branch of the UPR, including the genes *BIP*, *IRE1α*, and *XBP1*. Upon induction of ER stress in neuronal cells, miR-34a-5p overexpression impacts the resulting UPR via a significant reduction in IRE1α and XBP1s that in turn leads to decreased viability, increased cytotoxicity and caspase activity. The possibility to modify the UPR signaling pathway by a single miRNA that targets central genes of the IRE1α branch offers new perspectives for future therapeutic approaches against neurodegeneration.

## 1. Introduction

Accumulation of misfolded proteins is a hallmark of many diseases, including various cancer types [1] and neurodegenerative diseases like Alzheimer’s [2,3], Parkinson’s [3,4] and Huntington’s disease [5]. The accumulation of unfolded or misfolded proteins in the ER lumen, referred to as ER stress, induces apoptosis as part of the unfolded protein response (UPR) [6]. In mammalian cells, the UPR signaling pathway is mediated by three ER membrane stress sensors, including inositol requiring kinase 1α (IRE1α), activating transcription factor 6 (ATF6), and protein kinase RNA-like ER kinase (PERK) [7]. The activation of the PERK branch causes a rapid decrease in protein translation, while activation of IRE1α (inositol requiring kinase 1α) and ATF6 (activating transcription factor 6) results in a transcriptional response by increasing the expression of chaperones [8]. The IRE1α branch is the most conserved of these signaling pathways and has been targeted in many disease-specific therapeutic approaches [9].

Therapeutic interventions aiming at the modulation of cellular pathways can be obtained by microRNAs (miRNAs), which are small, non-coding RNAs that regulate gene expression on the posttranscriptional level by binding to the 3′untranslated regions (UTR) and, in fewer instances, to 5′ UTRs or open reading frames (ORFs) of their target mRNA [10,11,12,13]. However, miRNA binding sites in 5′ UTRs and ORFs often exhibit only modest effects on posttranscriptional regulation of the target mRNA [14].

MiRNAs qualify themselves as efficient modulators of cellular pathways, in particular by the fact that each miRNA may regulate the translation of an extended number of different genes.

Many diseases, including neurodegenerative diseases, are associated with aberrant miRNA expression in affected tissues and body fluids [15,16,17]. In several neurodegenerative diseases, miR-34a-5p has been previously described as deregulated. Specifically, up-regulation of miR-34a-5p has been found in blood samples of Alzheimer’s disease patients as well as in affected brain areas of Alzheimer’s and Parkinson’s disease patients [18,19,20] Here, we set out to analyze the role of miR-34a-5p on the UPR signaling pathway in neuronal cells to identify efficient ways for modulating cellular UPR stress response to reconstitute cell homoeostasis and to counteract neurodegeneration.

## 2. Materials and Methods

### 2.1. Cell Lines

HEK293T and SH-SY5Y cell lines were obtained from the German collection of microorganisms and cell cultures (DSMZ). HEK293T was cultured in Dulbecco’s Modified Eagle Medium (Life Technologies, Carlsbad, CA, USA) supplemented with 10% fetal bovine serum (FBS; Biochrom, Berlin, Germany), penicillin (100 U/mL) and streptomycin (100 μg/mL) at 37 °C and 5% CO_2_. 

The human neuroblastoma cell line SH-SY5Y was grown in Dulbecco’s Modified Eagle Medium (Life Technologies, Carlsbad, CA, USA) containing 20% fetal bovine serum (FBS; Biochrom, Berlin, Germany), penicillin (100 U/mL) and streptomycin (100 μg/mL) at 37 °C and 5% CO_2_.

### 2.2. In-Silico-Based Target Selection for miR-34a

To identify and to validate possible miR-34a-5p target genes, we combined an in-silico-based and experimental approach as it has been previously described by Hart et al. [21]. Therefore, we performed in-silico target prediction using miRWalk2.0 [22], focusing only on putative target genes that were predicted by at least four independent algorithms. Next, we performed a pathway enrichment analysis by GeneTrail2 (Table S1) [23]. We selected the gene ontology pathway IRE1α-mediated unfolded protein response (GO:0036498) and conducted a protein–protein association analysis with the STRING database version 11 [24] with an interaction score ≥ 0.4 to determine the specific role of miR-34a-5p in the IRE1α branch of the UPR. 

### 2.3. Generation of 3′UTR Reporter Constructs

To validate miR-34a-5p interactions with the predicted target genes, we cloned the 3′UTR of *BIP*, *IRE1α* and *XBP1* into the pMIR-RNL-TK vector. In detail, we amplified the nucleotides 564 to 1688 of *BIP* 3′UTR (NM_005347.4), nucleotides 3907 to 4829 of *IRE1α* 3′UTR (NM_001433.4) and nucleotides 11 to 930 of *XBP1* 3′UTR (NM_005080.3) using sequence-specific primers (Table S2) and cloned them into the multiple cloning site of the pMIR-RNL-TK expression plasmid by using SpeI and SacI restriction sites. Mutation of the miR-34a-5p binding sites in the 3′UTR of the respective genes was accomplished by using overlap extension PCR with specific primer pairs.

### 2.4. Dual-Luciferase Reporter Assay

For validation of the predicted targets with dual-luciferase reporter assay, HEK293T was seeded in 24-well plates with a density of 6 × 10^4^ cells per well. After 24 h, cells were transfected with 0.2 µg reporter gene construct and 0.8 µg miR-34a-5p expression plasmid using PolyFect transfection reagent (Qiagen, Hilden, Germany) according to the manufacturer’s recommendations. At 48 h after transfection, cells were lysed and dual-luciferase reporter assay was performed following the manufacturer’s instructions (Promega, Mannheim, Germany). Luciferase assays were performed in duplicates of three independent experiments.

### 2.5. Tunicamycin Treatment

To induce ER stress in SH-SY5Y, cells were treated with tunicamycin (Sigma Aldrich, Munich, Germany). For western blotting experiments, cells were treated with 5 µg/mL tunicamycin for 4 h. For time-lapse experiments, cells were treated with 1 µM tunicamycin for 8, 24 and 48 h. Control cells were treated with DMSO.

### 2.6. Western Blot

To determine the effects of miR-34a-5p on the protein level of the predicted targets, western blotting was performed. Therefore, HEK293T or SH-SY5Y were seeded with 2.5 × 10^5^ cells per well in six-well plates. The following day, HEK293T cells were transfected either with pSG5 vector or pSG5-miR-34a expression plasmid by using PolyFect Transfection Reagent (Qiagen, Hilden, Germany) according to the manufacturer’s protocol. Following the manufacturer’s instructions, SH-SY5Y cells were transfected with hsa-miR-34a-5p miScript miRNA Mimic or AllStar Negative Control (ANC) by using HiPerFect Transfection Reagent (Qiagen, Hilden, Germany). At 48 h after transfection, cells were either directly harvested or treated with 5 µg/mL tunicamycin for an additional 4 h before harvesting. Proteins were isolated with 2× lysis buffer (130 mM Tris/HCl, 6% SDS, 10% 3-mercapto-1,2-propandiol, 10% glycerol) by sonification. A 15 µg quantity of whole-cell protein extract was separated by SDS-PAGE on Mini-Protean^®^ TGX Stain-Free^TM^ Precast Gels (Bio-Rad Laboratories Inc., Hercules, CA, USA) and electroblotted on a nitrocellulose membrane (Whatman, GE Healthcare, Freiburg, Germany). To detect the proteins of interest, the antibodies anti-BiP monoclonal rabbit (3177S), anti-IRE1α monoclonal rabbit (3294S) and anti-XBP1s monoclonal rabbit (12782S) from Cell Signaling Technology (Danvers, MA, USA) were used. Anti-GAPDH monoclonal rabbit antibody (14C10, Cell Signaling Technology, Danvers, USA) or anti-β-Actin monoclonal mouse antibody (AC-15, Sigma Aldrich, Munich, Germany) was used as the endogenous control. The secondary antibodies used were obtained from Sigma Aldrich (Sigma Aldrich, Munich, Germany). Western blot analyses were performed in four independent experiments.

### 2.7. RNA-Isolation, Quantitative Real-Time PCR and Northern Blot

Total RNA was isolated using the miRNeasy Mini Kit (Qiagen, Hilden, Germany) according to the manufacturer’s protocol after cell lysis with Qiazol (Qiagen, Hilden, Germany). A 150 ng quantity of total RNA was applied for reverse transcription using the miScript PCR System (Qiagen, Hilden, Germany). qRT-PCR was performed with QuantiTect Primer Assay (Qiagen) on the StepOnePlus Real-Time PCR System (Applied Biosystems, Foster City, United States) with a specific primer for hsa-miR-34a-5p and *BIP*. The experiments were done in independent triplicates. Statistical significance was calculated by student’s t-test. Northern blotting was performed with specific radiolabeled probes against hsa-miR-34a-5p (TGGCAGTGTCTTAGCTGGTTGTCCTGTCTC) as described previously by our group [25].

### 2.8. Cell Viability and Cytotoxicity Assays

SH-SY5Y cells were seeded with 4 × 10^5^ cells per well in six-well plates. The next day, cells were transfected with hsa-miR-34a-5p miScript miRNA Mimic or AllStars Negative Control (ANC) using HiPerFect Transfection Reagent (Qiagen, Hilden, Germany). At 24 h after transfection, cells were re-plated with 1.8–2.5 × 10^4^ cells per well in opaque-walled 96-well plates. After an additional 24 h, cells were treated with 1 µM tunicamycin or DMSO as control for 8 h, 24 h and 48 h. Luciferase-based CellTiterGlo 2.0, CytotoxGlo and Caspase3/7 Glo assays (Promega, Mannheim, Germany) were applied according to the manufacturer’s instructions to determine cell viability, cytotoxicity and caspase activity. Luciferase activity was measured with a GloMax navigator microplate luminometer (Promega, Mannheim, Germany). The experiments were done in triplicate in two independent experiments. As control, ANC and miR-34a-5p-transfected cells were treated with DMSO. Cell viability and caspase activity were calculated by normalizing the received luminescence values to the control transfected cells treated with DMSO for each time point. The relative number of dead cells was calculated by normalizing the luminescence derived from the dead cell protease activity to the protease activity of total cells after cell lysis. Statistical significance was calculated with a two-tailed paired t-test. 

### 2.9. Quantification and Statistical Analysis

Statistical significance was analyzed with GraphPad Prism 7.04 (GraphPad Software, La Jolla, USA) using a student’s t-test as well as a two-tailed paired t-test. Western blots were quantified using Image Lab Software Version 5.2.1 (Bio-Rad Laboratories Inc., Hercules, CA, USA). 

## 3. Results

### 3.1. miR-34a-5p Directly Targets IRE1α Branch of the UPR

**Figure 1:**
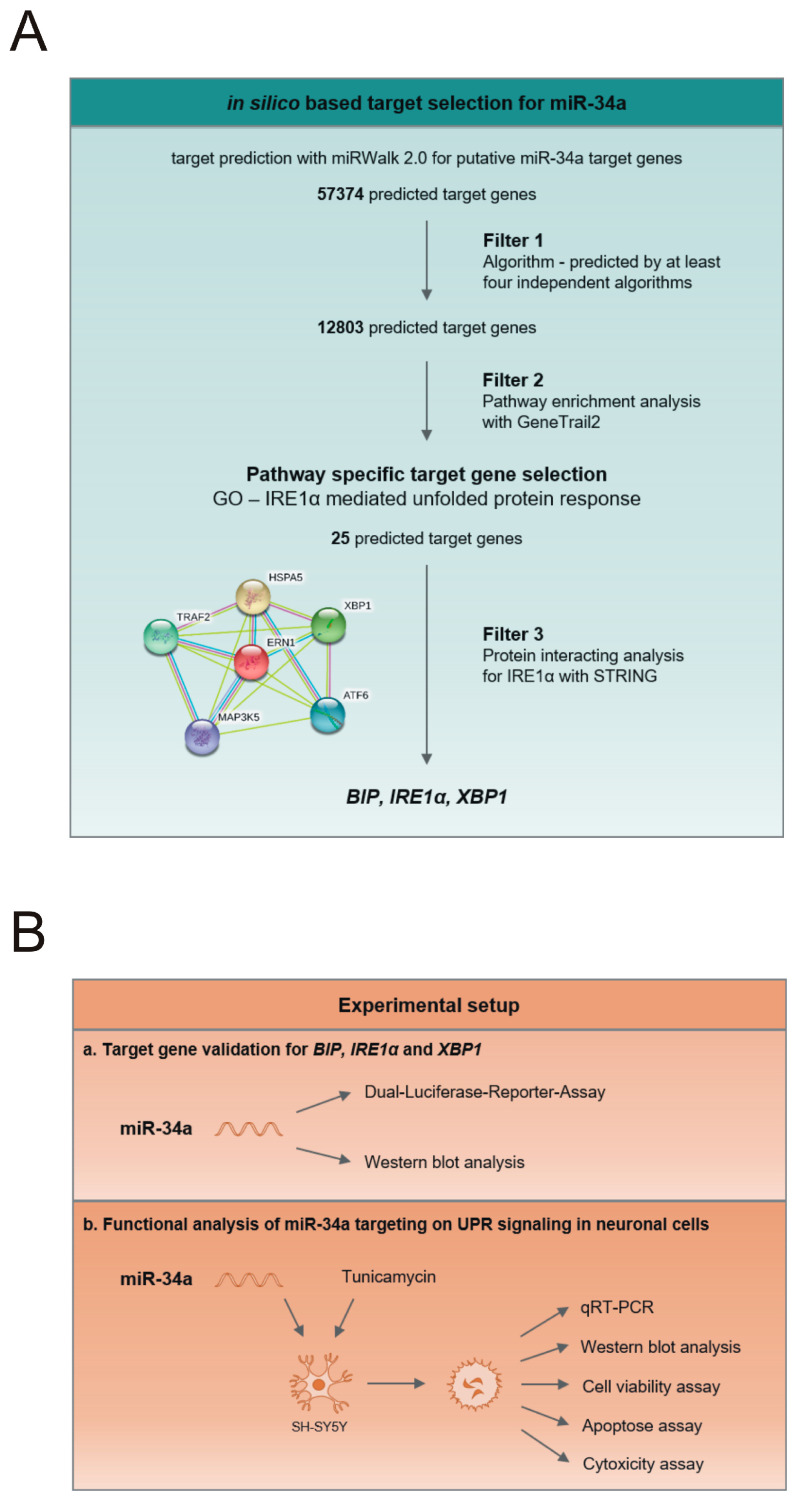
Paper workflow. (**A**) in-silico-based target selection for miR-34a, including protein interaction analysis by STRING. Putative miR-34a target genes were predicted by an in-silico-based approach and computationally filtered for IRE1α-mediated unfolded protein response resulting in the three target genes *BIP*, *IRE1α* and *XBP1*. STRING analysis: magenta lines: experimentally determined protein interactions; blue lines: known interactions from curated databases; yellow lines: text mining. (**B**) experimental setup. The target genes *BIP*, *IRE1α* and *XBP1* for miR-34a-5p were experimentally validated, and the effect of miR-34a targeting was analyzed by functional analysis.

To analyze the functional role of miR-34a in the UPR signaling pathway, we employed an in-silico-based target selection workflow (Figure 1A). We conducted a target prediction analysis by miRwalk 2.0 for putative miR-34a target genes [22]. We focused on canonical target sites that had a minimum seed length of 7 nt and were predicted by at least four independent algorithms. Next, we applied two additional filter steps on the predicted target genes. First, we performed a pathway enrichment analysis with GeneTrail2 [23]. Despite EIF2A, a member of the PERK branch, which exhibited a 7mer-A1 binding site, only target genes in the IRE1α branch harbored canonical miR-34a binding sites. Therefore, we focused on the gene ontology pathway “IRE1α-mediated unfolded protein response”, representing the most conserved branch of UPR signaling. This pathway revealed 25 putative target genes of miR-34a associated with UPR (GO:0036498; Figure S1, Table S1).

Second, to confine the number of potential target genes and unravel their functional as well as physical interactions in the IRE1α branch, we performed a protein–protein association analysis for IRE1α with the STRING database (Figure 1A). We found protein interactions of IRE1α (also termed ERN1) with BiP (also termed HSPA5), which were experimentally confirmed, as well as an interaction of IRE1α with XBP1 indicated by curated databases. As a result of this selection step, we obtained proteins of the IRE1α pathway that were not only miR-34a targets but were also interacting within this pathway. To verify the impact of multiple-miR-34a-5p targeting, we employed an experimental setup consisting of target gene validation as well as functional analysis (Figure 1B).

**Figure 2:**
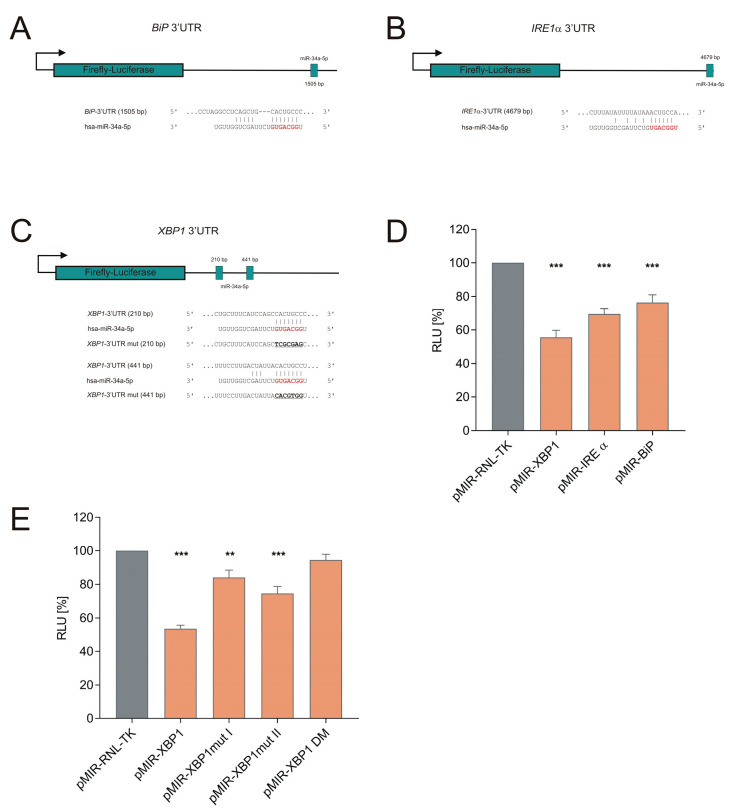
miR-34a targets the IRE1α branch of the UPR. (**A**–**C**) Schematic overview of *BIP* (**A**), *IRE1α* (**B**) and *XBP1* (**C**) 3′UTRs with miR-34a-5p binding sites. 3′UTR sequences of *BIP*, *IRE1α* and *XBP1* were cloned into pMIR-RNL-TK reporter plasmids. The locations of the miR-34a-5p-predicted binding sites are shown schematically in the 3′UTR of the target genes as well as in the sequence of the 3′UTR (red). Sequences that are mutated to replace the miR-34a-5p binding site are underlined. (**D**) Dual-luciferase reporter assays for *BIP*, *IRE1α* and *XBP1*. HEK293T cells were transfected with reporter gene construct as well as a miR-34a expression plasmid in the indicated combinations. Luciferase activity was measured 48 h post transfection. The RLUs were normalized to the RLU received with the empty pMIR-RNL-TK reporter plasmid co-transfected with a miR-34a expression plasmid. (**E**) Mutagenesis assay for *XBP1*. HEK293T cells were transfected with reporter gene constructs for *XBP1* with mutated miR-34a binding sites as well as miR-34a expression plasmid. Luciferase activity was measured 48 h post transfection. The RLUs were normalized to the RLU received with the empty pMIR-RNL-TK reporter plasmid co-transfected with miR-34a expression plasmid. The experiments were performed as three biological replicates, each in duplicate. Data represent mean ± SEM (* = *p* ≤ 0.05, ** = *p* ≤ 0.01, *** = *p* ≤ 0.001).

The *BIP* and *IRE1α* 3′UTRs harbored one binding site, respectively, whereas the gene *XBP1* contained two binding sites within its 3′UTR (Figure 2A–C). To experimentally validate the predicted interactions between miR-34a-5p and the 3′UTR of the target genes, we cloned the 3′UTR sequences of the targets into pMIR-RNL-TK reporter plasmids, co-transfected these plasmids together with a miR-34a-5p expression plasmid in HEK293T cells, and measured the luciferase activity after 48 h. The dual luciferase assays showed a reduction in the relative luciferase activity (relative lights units, RLU) to 76% (*p* ≤ 0.001) for the *BIP* reporter plasmid pMIR-RNL-TK *BIP* 3′UTR, to 69% (*p* ≤ 0.001) for the *IRE1α* reporter plasmid pMIR-RNL-TK *IRE1α* 3′UTR, and to 56% (*p* ≤ 0.001) for the *XBP1* reporter plasmid pMIR-RNL-TK *XBP1* 3′UTR (Figure 2D), each compared to control reporter plasmid pMIR-RNL-TK.

To determine the role of the two binding sites identified in the 3′UTR of the *XBP1* gene, we separately mutated both miRNA binding sites of this gene. Mutation of the first binding site using the recombinant pMIR-RNL-TK *XBP1* 3′UTR mut I reduced the luciferase activity to 84% and mutation of the second binding site using the recombinant pMIR-RNL-TK *XBP1* 3′UTR mut II to 74% (Figure 2E). Compared to wild-type *XBP1* 3′UTR, the mutations showed a significantly increased luciferase activity for the mutated reporter plasmids (*p* ≤ 0.001 for pMIR-RNL-TK *XBP1* 3′UTR mut I and *p* ≤ 0.01 for pMIR-RNL-TK *XBP1* 3′UTR mut II). This shows that the mutation of either of the two *XBP1* binding sites is sufficient to increase the luciferase activity as compared to the wild type *XBP1* sequence. The mutation of both binding sites together using the recombinant pMIR-RNL-TK *XBP1* 3′UTR DM yielded no significant reduction of the luciferase activity compared to the control reporter plasmid pMIR-RNL-TK, but, again, significantly increased luciferase activity compared to the wild type *XBP1* 3′UTR (*p* ≤ 0.001). Together, these results indicate that both binding sites contribute to the biological effects of miRNA binding within the *XBP1* 3′UTR.

**Figure 3:**
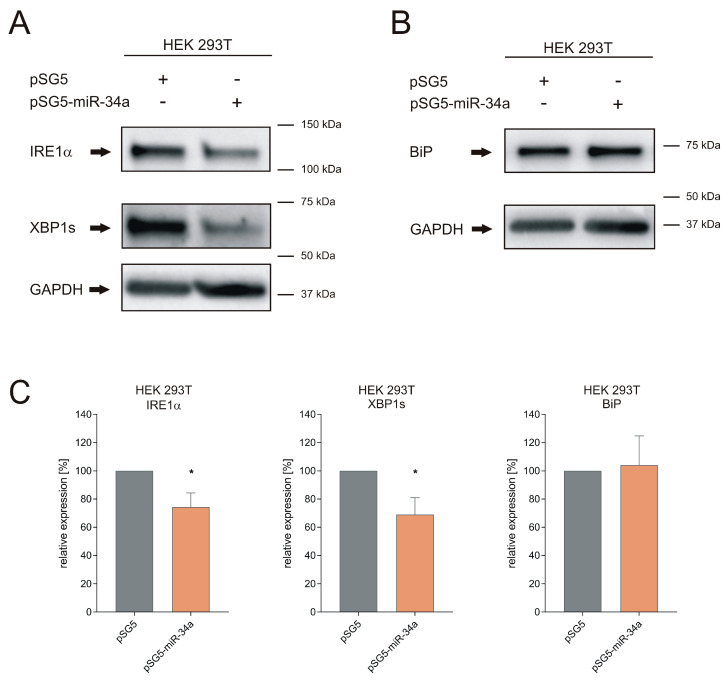
Detection of BiP, IRE1α and XBP1s endogenous protein after miR-34a transfection. (**A**,**B**) Representative western blots for IRE1α, XBP1s and BiP. HEK293T cells were transfected with pSG5 or pSG5-miR-34a expression plasmid for 48 h. Changes in protein level were detected by specific antibodies against IRE1α, XBP1s and BiP. GAPDH served as an endogenous control. (**C**) Quantification of IRE1α, XBP1s and BiP protein level. Western blots were quantified by densitometry by Image Lab Software and normalized to GAPDH. Data represent mean ± SEM of four independent experiments (* = *p* ≤ 0.05).

To further validate the results of the luciferase assays, we tested the impact of miR-34a-5p overexpression on the endogenous protein level of the miRNA target genes *BIP*, *IRE1α*, and *XBP1s* in HEK293T cells. After transfection of HEK293T cells with pSG5-miR-34a expression plasmid or pSG5 control plasmid, we determined changes in protein levels with specific antibodies in four independent experiments, each using GAPDH as an endogenous control. Figure 3A,B show one representative western blot for each target. Overexpression of miR-34a-5p led to a decrease to 74% (*p* ≤ 0.05) of the IRE1α protein levels and to 69% (*p* ≤ 0.05) of the levels of XBP1s, which is the active form of XBP1 protein (Figure 3C). The endogenous levels of the BiP protein were not significantly affected. 

### 3.2. miR-34a-5p Overexpression Overwrites the Effect of ER Stress on IRE1α and XBP1s Proteins in Neuronal Cells

**Figure 4:**
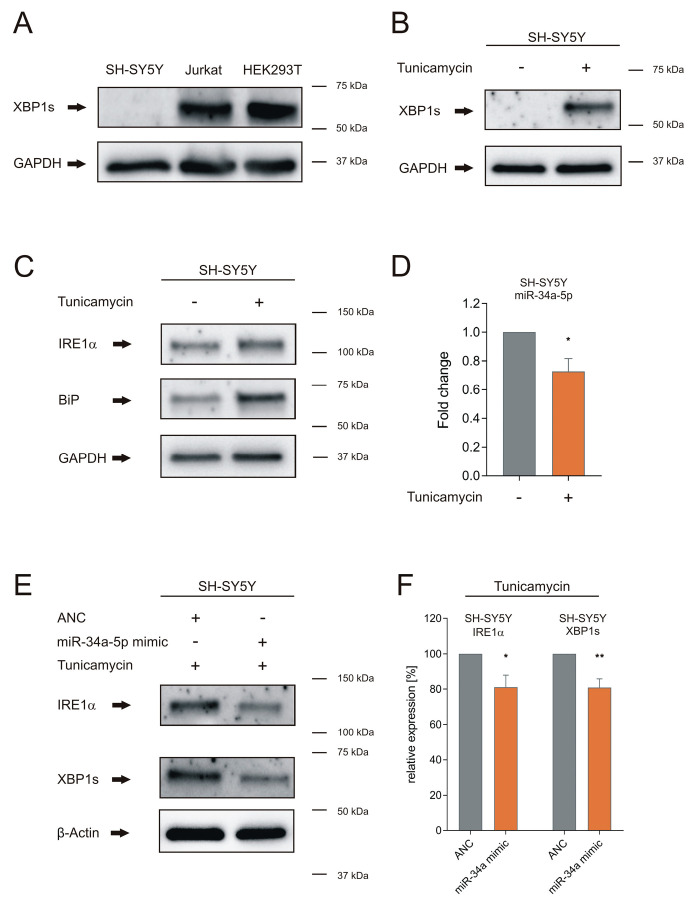
Induction of the UPR by tunicamycin in neuronal SH-SY5Y cells. (**A**) XBP1s endogenous protein level in SH-SY5Y, Jurkat and HEK293T cells. Proteins of SH-SY5Y, Jurkat and HEK293T cells were isolated, and western blotting was performed using XBP1s-specific antibody. GAPDH served as the endogenous control. (**B**) Endogenous XBP1s protein after tunicamycin treatment in SH-SY5Y cells. SH-SY5Y cells were treated with tunicamycin for 4 h, and western blotting was performed using XBP1s-specific antibody. GAPDH served as endogenous control. (**C**) Detection of endogenous protein levels of IRE1α and BiP after tunicamycin treatment. SH-SY5Y cells were treated with tunicamycin for 4 h and western blotting was performed using IRE1α- and BiP-specific antibodies. GAPDH served as the endogenous control. (**D**) Analysis of miR-34a-5p after tunicamycin treatment in SH-SY5Y cells by qRT-PCR. SH-SY5Y cells were treated with tunicamycin for 4 h. RNA was isolated and qRT-PCR was performed using miR-34a-5p-specific primer. Data represent mean ± SEM of three independent experiments (*p* ≤ 0.05). (**E**) Representative western blot of IRE1α and XBP1s protein levels in miR-34a-5p overexpressing SH-SY5Y cells after tunicamycin treatment. SH-SY5Y cells were transfected with AllStars Negative Control (ANC) or miR-34a-5p mimic. At 48 h post transfection, cells were treated with tunicamycin for an additional 4 h. Endogenous protein levels of IRE1α and XBP1s were analyzed by western blotting using specific antibodies against IRE1α and XBP1s. β-Actin served as the endogenous control. (**F**) Quantification of endogenous protein levels of IRE1α and XBP1 in miR-34a-5p-overexpressing SH-SY5Y cells after tunicamycin treatment. Western blots were quantified by densitometry by Image Lab Software and normalized to β-Actin. Data represent mean ± SEM of four independent experiments (* = *p* ≤ 0.05).

After having demonstrated the effect of miR-34a-5p on endogenous IRE1α and XBP1s in HEK293T cells, we further analyzed the role of miR-34a-5p targeting on UPR signaling in neuronal cells that were challenged to develop ER stress. As the cell model, we choose neuronal SH-SY5Y cells and treated them with tunicamycin for 4 h. In contrast to Jurkat and HEK293T, endogenous XBP1s was not detectable in control SH-SY5Y cells treated with DMSO, as shown by western blot analysis (Figure 4A). Upon the induction of ER stress by tunicamycin, we found increased levels of XBP1s as well as IRE1α and BiP protein and *BIP* mRNA in SH-SY5Y cells (Figure 4B,C and Figure S2). We also found significantly reduced levels of miR-34a-5p in tunicamycin-treated SH-SY5Y cells (*p* ≤ 0.05) (Figure 4D). We next tested whether the effects of the induced ER stress on XBP1s and on IRE1α could be overwritten by miR-34a-5p using specific antibodies against IRE1α and the spliced isoform of XBP1, XBP1s. To this end, we transfected SH-SY5Y cells with miR-34a-5p mimic or AllStars Negative Control (ANC) and treated them after 48 h with tunicamycin for 4 h. We found that overexpression of miR-34a-5p, which was verified by northern blotting (Figure S3), significantly reduced the protein levels of IRE1α and XBP1s to 81% in tunicamycin-treated SH-SY5Y cells (IRE1α: *p* ≤ 0.05, XBP1s: *p* ≤ 0.01), as shown by western blot analysis with specific antibodies (Figure 4E,F). With IRE1α and XBP1s being both central to the UPR, these results emphasize the role of miR-34a-5p for the UPR.

### 3.3. Effects of miR-34a-5p Overexpression on Viability of Neuronal Cells during ER Stress

**Figure 5:**
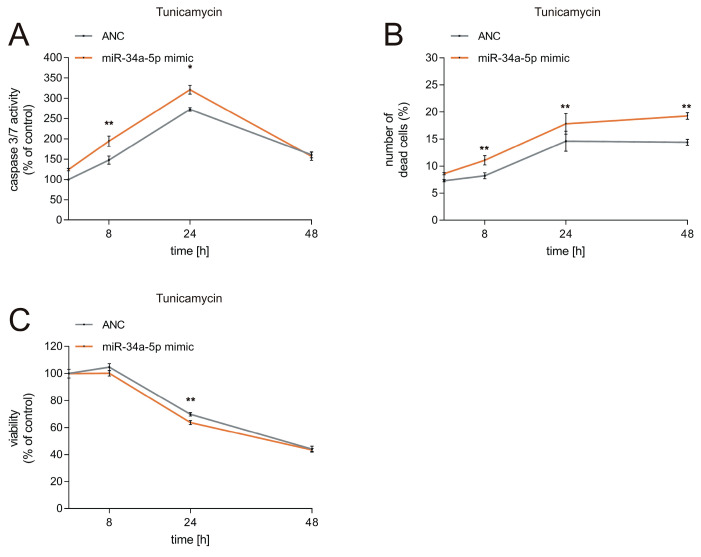
Functional effects of miR-34a-5p targeting on the UPR. (**A**–**C**) Results of the caspase 3/7 assay (**A**), cytotoxicity assay (**B**) and viability assay (**C**) in miR-34a-5p transfected SH-SY5Y cells treated with tunicamycin. SH-SY5Y cells were transfected with AllStars Negative Control (ANC) or miR-34a-5p mimic and treated with tunicamycin for the indicated times. Caspase 3/7 activity, cytotoxicity, and cell viability were measured by changes in luciferase activity. The experiments were done in triplicate in two independent experiments (* = *p* ≤ 0.05, ** = *p* ≤ 0.01).

To further test the functional effects of miR-34a-5p on the UPR in neuronal cells, we analyzed cell viability, caspase activity, and cytotoxicity as read out. The results demonstrate that caspase activity is significantly elevated in cells which undergo ER stress caused by tunicamycin (Figure 5A, Figure S4A). MiR-34a-5p overexpression in SH-SY5Y cells resulted in a 46% elevation of caspase activity measured 8 h after tunicamycin treatment (*p* ≤ 0.01) and in a 48% elevation measured 24 h after tunicamycin treatment (*p* ≤ 0.05), each compared to controls, i.e., tunicamycin-treated cells transfected by ANC (Figure 5A). Measurement of caspase activity 48 h after tunicamycin treatment showed no significant difference between cells transfected by miR-34a-5p and control transfected cells. In control cells treated with DMSO, miR-34a-5p overexpression led to a maximum increase in caspase activity of 20% as compared to control transfected cells (Figure S4A). As for cytotoxicity, miR-34a-5p overexpression in SH-SY5Y cells resulted in a 3% elevation of the number of dead cells measured 8 h after tunicamycin treatment (*p* ≤ 0.01), in a 15% elevation measured 24 h after tunicamycin treatment (*p* ≤ 0.01), and in an 18% elevation 48 h after tunicamycin treatment (*p* ≤ 0.01), again each compared to controls: i.e., cells transfected by ANC (Figure 5B). MiR-34a-5p overexpression in control SH-SY5Y cells treated with DMSO led to a maximum increase in the number of dead cells of 2%, 48 h following the tunicamycin treatment (Figure S4B). As for the viability, miR-34a-5p overexpression in SH-SY5Y cells resulted only in a slightly decreased viability measured 8 h after tunicamycin treatment but in a 36% reduction of viability measured 24 h after tunicamycin treatment. Compared to control transfected cells treated with tunicamycin, miR-34a-5p led to significantly reduced cell viability 24 h after tunicamycin treatment (*p* ≤ 0.01) (Figure 5C). Viability measurement 48 h after tunicamycin treatment showed no significant difference between cells transfected with miR-34a-5p and cells transfected with the control. In control cells treated with DMSO, overexpression of miR-34a-5p lead only to a slightly decreased viability, ranging from 1.3% to 3.7% over the indicated time (Figure S4C).

These results indicate that the most prominent effects of miR-34a-5p on cell viability and caspase activity occur 24 h after ER stress has been induced by tunicamycin. MiR-34a-5p overexpression affected the cytotoxicity most 48 h after induction of ER stress.

## 4. Discussion

We showed that miR-34a-5p, which is upregulated in the brains of Alzheimer’s and Parkinson’s disease patients, directly targets the genes *BIP* (binding immunoglobulin protein), *IRE1α* (inositol-requiring enzyme 1 α), and *XBP1* (X-box binding protein 1), all of which are part of the IRE1α branch of the UPR. Although miR-34a-5p targets the 3′UTR of *BIP*, western blot analysis revealed no effect of miR-34a-5p overexpression on BiP. As endogenous BiP protein level is under the control of selective translation by phosphorylated EIF2A, which is a predicted target gene of miR-34a, we assume a second layer of UPR regulation by miR-34a-5p via the PERK branch [26]. We were, however, able to confirm *IRE1α* as a direct target gene of miR-34a-5p both by luciferase assays and by western blot analysis. IRE1α exhibits two enzymatic domains including a serine/threonine–protein kinase domain and an endoribonuclease (RNase) domain. Upon dissociation of BiP, IRE1α undergoes a conformational change resulting in activation of the kinase domain and transautophosphorylation [27,28]. Subsequently, the RNase domain performs unconventional splicing of the *XBP1* mRNA, leading to excision of a 26-nt long intron. The resulting frame shift in the coding region of the *XBP1* mRNA gives rise to the active *XBP1s* form, which functions as a transcription factor for UPR-associated genes [29]. Posttranscriptional regulation of the *XBP1* mRNA has been previously reported for different miRNAs, leading to similar functional effects as shown by our results [30,31]. Here, we report that miR-34a directly targets the IRE1α branch of the UPR by regulating the two key components, XBP1 and IRE1α, underlining a more complex regulatory network. 

**Figure 6:**
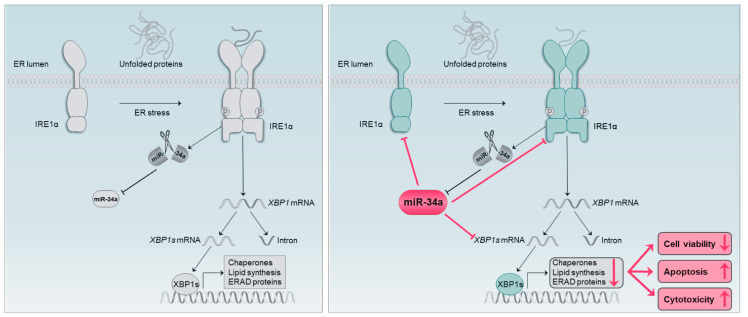
The hypothesized role of miR-34a-5p in the IRE1α branch of UPR signaling. Left panel: the previously described interaction of miR-34a-5p and the IRE1α branch. Right panel: novel findings about the role of miR-34a-5p in the IRE1α branch based on our results.

Towards a deeper understanding of miR-34a-5p targeting on UPR signaling in neurodegeneration, we chose neuronal SH-SY5Y cells that showed no basal level of the active XBP1 protein (XBP1s). ER stress and subsequent UPR activation were induced by tunicamycin, which is widely used for induction of the UPR and is also applied in in-vitro models of Parkinson’s disease [32]. Tunicamycin inhibits N-linked protein glycosylation, leading to accumulation of misfolded proteins in the ER [33], a hallmark of several neurodegenerative diseases [34]. The tunicamycin-induced expression of active XBP1s, IRE1α, and BiP protein, as well as increased *BIP* mRNA in SH-SY5Y cells, indicates effective induction of ER stress. Moreover, we found that miR-34a-5p is significantly reduced upon tunicamycin treatment (Figure 4D), as also described by Sun et al. [35]. This observation can be explained by a study of Upton et al., which reported a rapid decay of select microRNAs, including miR-34a, by a sustained IRE1α RNase activity [36]. The study of Upton et al., together with our results, could point to a positive feedback loop of IRE1α and miR-34a-5p, which needs further experimental validation. Under physiological conditions, miR-34a-5p might possibly repress over-action of the IRE1α–XBP1 pathway. We hypothesize that the ER stress in neurodegeneration impairs the UPR by this feedback loop, in which miR-34a-5p plays a central role. This assumption is supported by our results showing that simulating central molecular aspects of neurodegeneration by inducing ER stress and overexpression of miR-34a-5p leads to significantly reduced IRE1α and XBP1s protein levels, reduced cell viability, increased cytotoxicity and increased caspase activity, all of which are indicative of impaired UPR signaling (Figure 6). Consistent with these results is the idea of a neuroprotective role for XBP1s in Alzheimer’s and Parkinson’s disease [37,38]. In summary, we hypothesize that the overexpression of miR-34a-5p in affected brain regions of Alzheimer’s and Parkinson’s disease patients could contribute to neurodegeneration by targeting IRE1α and XBP1. As a consequence, UPR signaling would be affected and the accumulation of misfolded proteins could be intensified.

Our study underlines the central role of miR-34a-5p in the regulation of the IRE1α branch by targeting IRE1α as well as XBP1s, thereby offering a way of modulating UPR signaling.

## Supplementary Materials

The following are available online at https://www.mdpi.com/2073-4409/9/6/1442/s1, Figure S1: similarity heatmap for IRE1-mediated unfolded protein response (GO:0036498) by GeneTrail2 analysis. Figure S2: induction of UPR signaling in neuronal SH-SY5Y (A) and HEK293T cells (B). Figure S3: analysis of miR-34a-5p overexpression in SH-SY5Y cells by northern blot. Figure S4: results of the caspase 3/7 assay (A), cytotoxicity assay (B) and viability assay (C) in miR-34a-5p-transfected SH-SY5Y cells treated with DMSO. Table S1: results of the GeneTrail2 analysis for similar categories to IRE1-mediated unfolded protein response (GO:0036498). Table S2: sequences of cloning and mutagenesis primer.
