# Supplementary material for: Induction of the Endoplasmic-Reticulum-Stress Response: MicroRNA-34a Targeting of the IRE1α-Branch
Source: Cells. 2020 Jun 10;9(6):1442. doi: 10.3390/cells9061442 (PMC7348704; doi:10.3390/cells9061442)
Supplement: Supplementary file 1 [file cells-09-01442-s001.zip › Supplement/Supplementary Legends.docx]

**SUPPLEMENTARY FIGURE LEGENDS**

**Figure S1: Similarity Heatmap for IRE1 mediated unfolded protein response (GO:0036498) by GeneTrail Analysis.** Predicted miR-34a target genes were analyzed by GeneTrail2 and the category IRE1 mediated unfolded protein response was selected for further target gene selection.

**Figure S2: Induction of UPR signaling in neuronal SH-SY5Y (A) and HEK293T cells (B).** SH-SY5Y (A) and HEK293T (B) cells were treated with Tunicamycin for 4 hours. RNA was isolated and qRT-PCR was performed using *BIP* specific primer. Data represent mean ± SEM of three independent experiments (** = p ≤ 0.01; (*** = p ≤ 0.001).

**Figure S3: Analysis of miR-34a-5p overexpression in SH-SY5Y cells by Northern Blot**. SH-SY5Y were transfected with ANC or miR-34a-5p mimics. 48 hours post transfection, RNA was isolated and northern blotting was performed using specific radiolabeled probe against hsa-miR-34a-5p.

**Figure S4: Functional effects of miR-34a-5p targeting in control cells.** A-C: Results of the caspase 3/7 assay (A), cytotoxicity assay (B) and viability assay (C) in miR-34a-5p transfected SH-SY5Y cells treated with DMSO. SH-SY5Y cells were transfected with all stars negative control (ANC) or miR-34a-5p mimic and treated with DMSO for indicated times. Caspase 3/7 activity, cytotoxicity as well as cell viability was measured by changes in luciferase activity. The experiments were done in triplicates in two independent experiments (* = p ≤ 0.05, ** = p ≤ 0.01).

**SUPPLEMENTARY TABLE LEGENDS**

**Table S1: Results of the GeneTrail2 analysis for similar Categories to IRE1 mediated unfolded protein response (GO:0036498).**

**Table S2: Sequences of cloning and mutagenesis primers.** Restriction sites are underlined. Mutated binding sites are underlined and bold.
