# Supplementary figures and images for: Induction of the Endoplasmic-Reticulum-Stress Response: MicroRNA-34a Targeting of the IRE1α-Branch
Source: Cells. 2020 Jun 10;9(6):1442. doi: 10.3390/cells9061442 (PMC7348704; doi:10.3390/cells9061442)

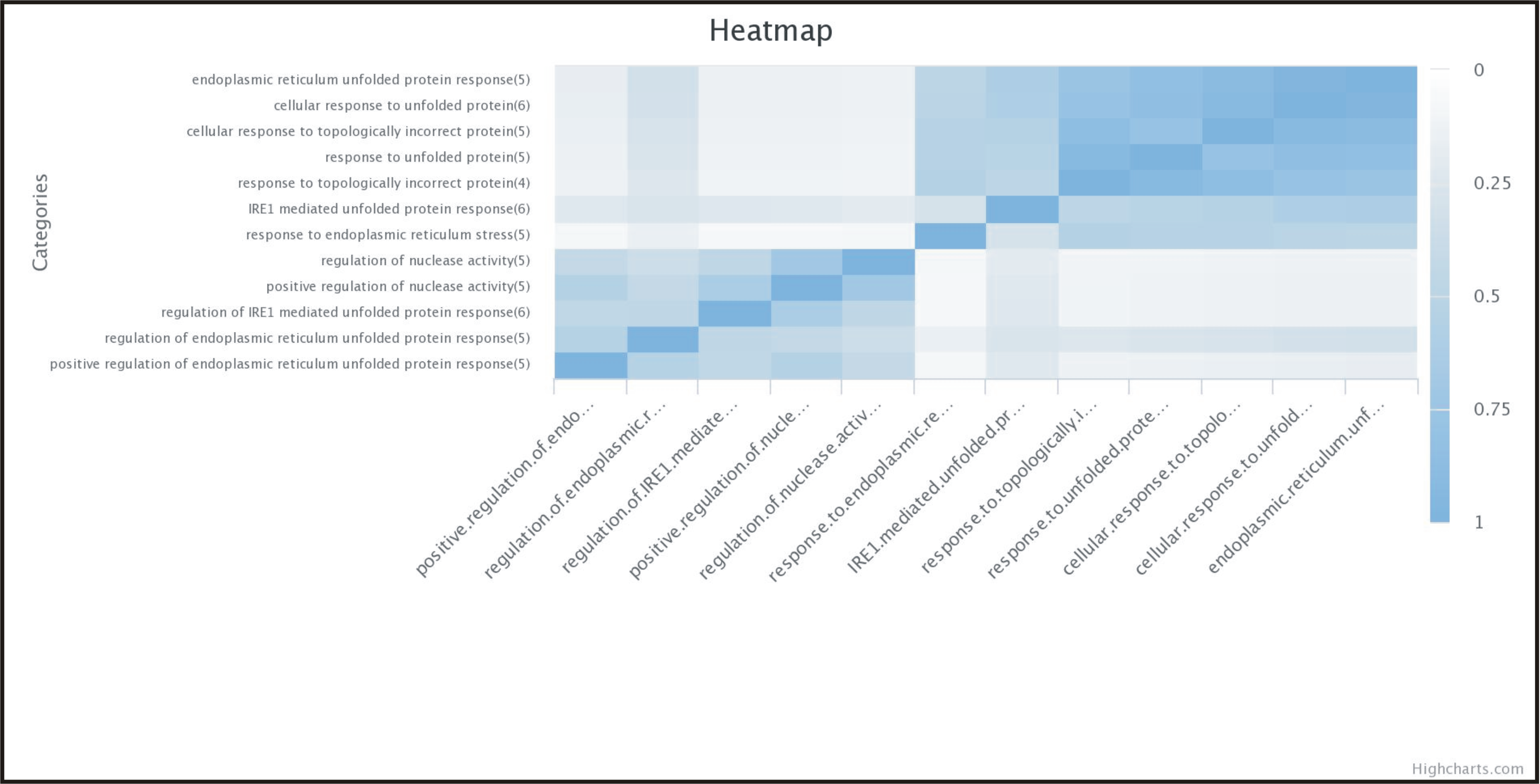

Supplement: Supplementary file 1 [file cells-09-01442-s001.zip › Supplement/SFigure 1.tif]

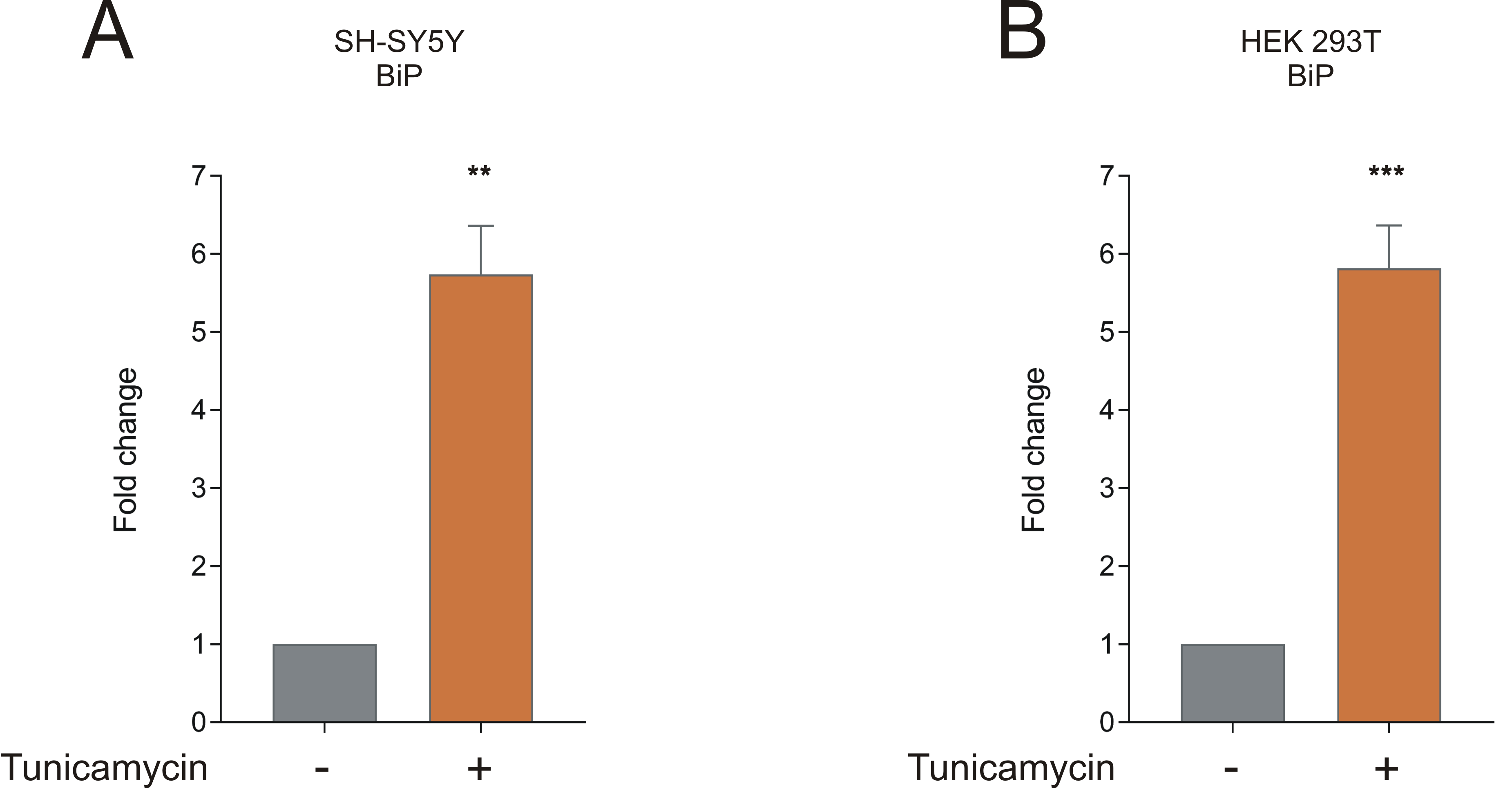

Supplement: Supplementary file 1 [file cells-09-01442-s001.zip › Supplement/SFigure 2.tif]

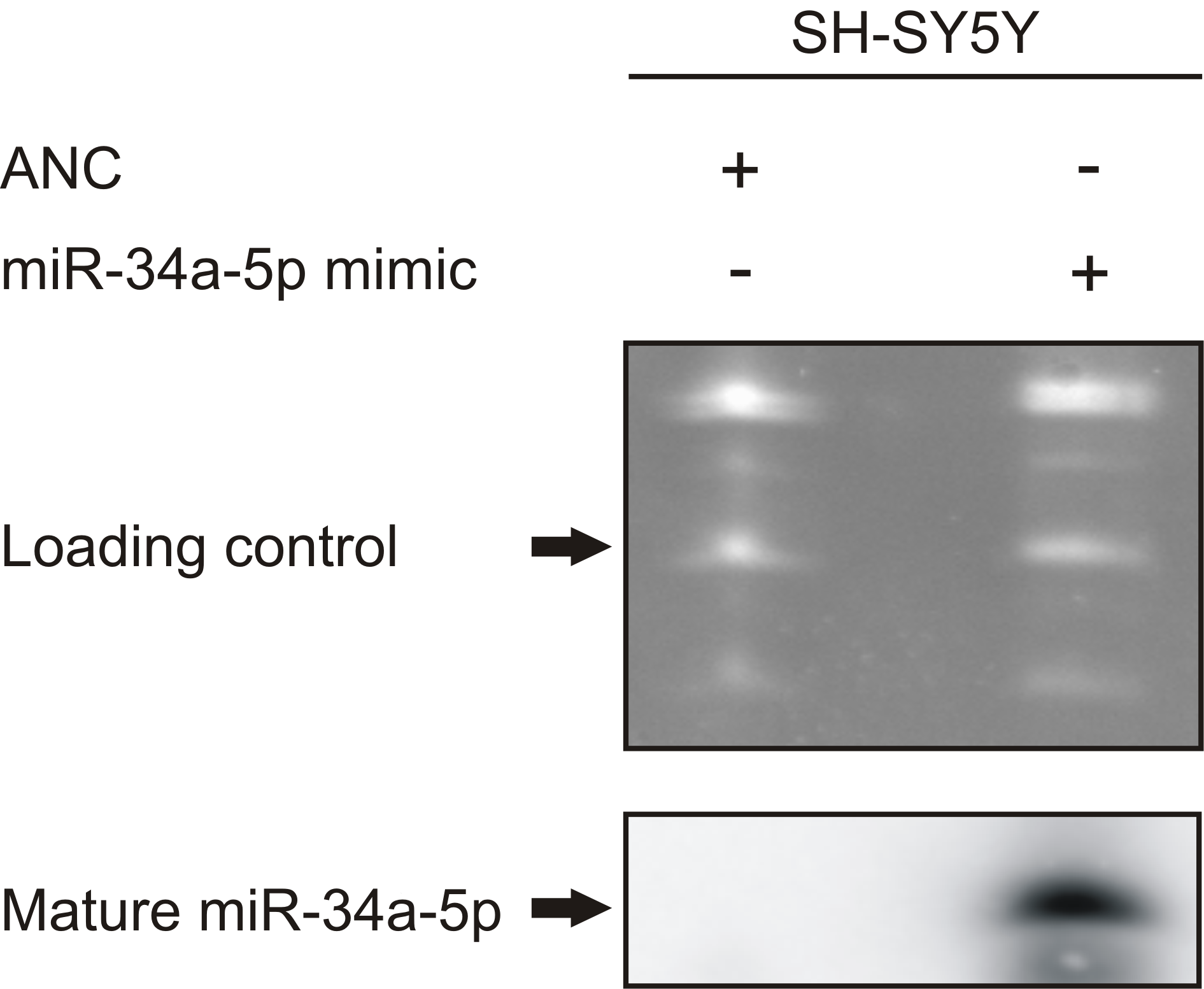

Supplement: Supplementary file 1 [file cells-09-01442-s001.zip › Supplement/SFigure 3.tif]

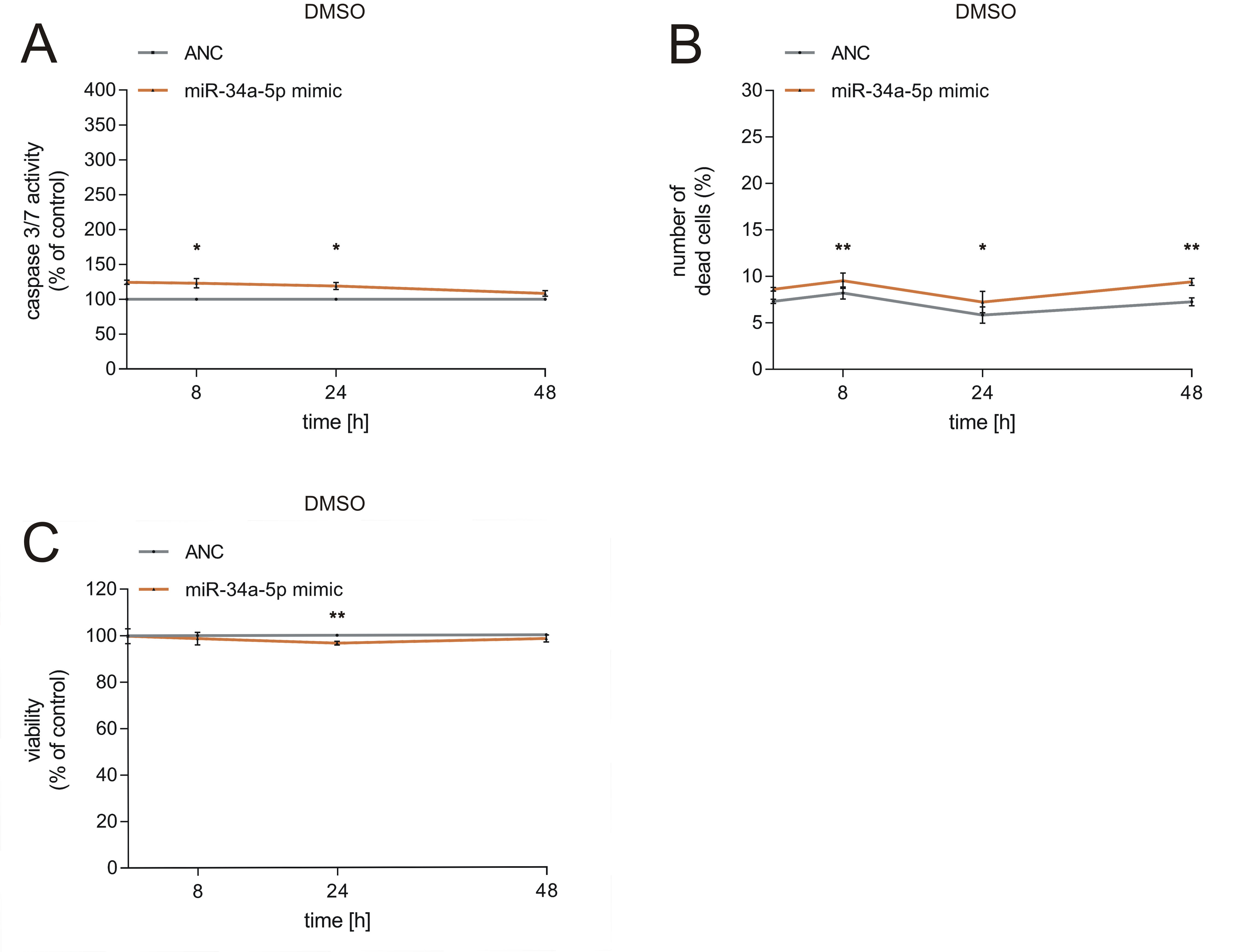

Supplement: Supplementary file 1 [file cells-09-01442-s001.zip › Supplement/SFigure 4.tif]
